# Increasing the Generalization of Supervised Fabric Anomaly Detection Methods to Unseen Fabrics

**DOI:** 10.3390/s22134750

**Published:** 2022-06-23

**Authors:** Oliver Rippel, Corinna Zwinge, Dorit Merhof

**Affiliations:** Institute of Imaging & Computer Vision, RWTH Aachen University, 52062 Aachen, Germany; corinna.zwinge@rwth-aachen.de (C.Z.); dorit.merhof@lfb.rwth-aachen.de (D.M.)

**Keywords:** supervised anomaly detection, automated visual inspection, fabric defect detection, model generalization

## Abstract

Fabric anomaly detection (AD) tries to detect anomalies (i.e., defects) in fabrics, and fabric AD approaches are continuously improved with respect to their AD performance. However, developed solutions are known to generalize poorly to previously unseen fabrics, posing a crucial limitation to their applicability. Moreover, current research focuses on adapting converged models to previously unseen fabrics in a post hoc manner, rather than training models that generalize better in the first place. In our work, we explore this potential for the first time. Specifically, we propose that previously unseen fabrics can be regarded as shifts in the underlying data distribution. We therefore argue that factors which reportedly improve a model’s resistance to distribution shifts should also improve the performance of supervised fabric AD methods on unseen fabrics. Hence, we assess the potential benefits of: (I) vicinal risk minimization (VRM) techniques adapted to the fabric AD use-case, (II) different loss functions, (III) ImageNet pre-training, (IV) dataset diversity, and (V) model architecture as well as model complexity. The subsequently performed large-scale analysis reveals that (I) only the VRM technique, AugMix, consistently improves performance on unseen fabrics; (II) hypersphere classifier outperforms other loss functions when combined with AugMix and (III) ImageNet pre-training, which is already beneficial on its own; (IV) increasing dataset diversity improves performance on unseen fabrics; and (V) architectures with better ImageNet performance also perform better on unseen fabrics, yet the same does not hold for more complex models. Notably, the results show that not all factors and techniques which reportedly improve a model’s resistance to distribution shifts in natural images also improve the generalization of supervised fabric AD methods to unseen fabrics, demonstrating the necessity of our work. Additionally, we also assess whether the performance gains of models which generalize better propagate to post hoc adaptation methods and show this to be the case. Since no suitable fabric dataset was publicly available at the time of this work, we acquired our own fabric dataset, called OLP, as the basis for the above experiments. OLP consists of 38 complex, patterned fabrics, more than 6400 images in total, and is made publicly available.

## 1. Introduction

Fabric anomalies (i.e., defects) have a strong economic impact, making their detection an essential aspect of fabric production [1]. However, anomaly detection AD in fabrics is still largely performed by human operators, and the outcome depends on the training, skill level, and fatigue of the personnel [2]. Even at peak performance, human operators are only capable of detecting 60–80% of defects [3,4], while simultaneously accounting for at least 10% of total labor costs [5]. Together, this calls for machine vision solutions that are capable of automated defect detection.

In context of automated fabric defect detection, semi-supervised methods (methods that require normal/defect-free data only [6,7]) are most commonly employed. The reason for this is that anomalies such as fabric defects are rare events and expensive to sample, whereas normal data are typically readily available. However, semi-supervised methods are currently limited to fabrics of low complexity (i.e., unimodal appearance) [8,9,10,11].

For fabrics of high complexity (i.e., multimodal appearance), supervised approaches that require both normal and anomalous data [6,7] are predominantly used. For example, classification, segmentation and object detection approaches have been successfully adapted to the fabric inspection task [12,13,14,15,16,17]. Moreover, supervised algorithms generally outperform their semi-supervised counterparts [18,19]. However, supervised methods suffer from a major drawback: They generalize poorly to fabrics unseen during model training [20,21] and therefore do not meet the industrial requirement for low changeover costs. Instead, defective and defect-free data must be collected and annotated for every new fabric, which is a tedious, time-consuming, and expensive process.

**Figure 1:**
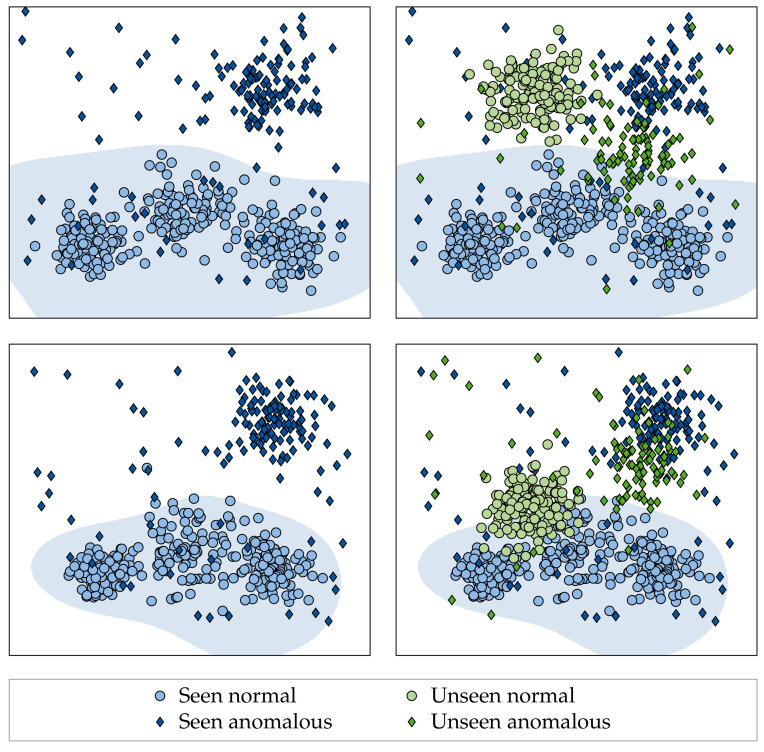
Toy problem demonstrating different ways generalization can manifest itself in multi-fabric AD. While both the poorly generalizing model (top left) and the better generalizing model (bottom left) achieve similar performance on the fabrics they were trained on, the performance on fabrics unseen during training may differ significantly. Comparing the two models, it becomes clear that the better generalizing model has a tighter clustering of normal data (and, correspondingly, a tighter decision boundary), a larger distance between anomalous and normal data clusters and maps the clusters of the unseen fabrics closer to their corresponding seen counterparts (i.e., anomalous to anomalous and normal to normal).

While algorithms have been proposed to tackle this limitation, current research focuses on adapting converged models to new fabrics in a post hoc manner [20,21]. It thereby disregards the potential of training models that generalize better to unseen fabrics in the first place (see Figure 1). In our work, we explore this potential for the first time in the context of supervised fabric AD/inspection, but note that related articles cover generalization for computer vision in natural images [22,23,24]. However, a clear academic consensus has not yet been established, and many research questions remain unanswered. Moreover, it has been shown recently that no proposed hypothesis/method consistently improves performance across different kinds of distribution shifts [22]. Together, this strongly demonstrates the necessity of our work, as we propose to view previously unseen fabrics as shifts in the underlying data distribution. Specifically, our contributions are as follows:

- We set up an exhaustive dataset containing 38 complex fabrics and more than 6400 images in total. The dataset is used to thoroughly validate all the findings of our work and is made publicly available at https://github.com/ORippler/OLP-dataset.
- We propose to view previously unseen fabrics as shifts in the underlying data distribution. We therefore argue that factors which reportedly improve a model’s resistance to distribution shifts [22,23,24,25] should also improve the generalization of supervised fabric AD methods to unseen fabrics and evaluate the potential benefits of: (I) VRM techniques adapted to the fabric AD use-case, (II) different loss functions, (III) ImageNet [26] pre-training, (IV) dataset diversity, and (V) model architecture as well as model complexity.
- We furthermore investigate whether better generalizing models are synergetic to post hoc adaptation methods such as [21], i.e., whether the performance on unseen fabrics can be further increased by applying post hoc adaptation methods.

## 2. Related Work

We give an overview of post hoc adaptation techniques in Section 2.1, followed by an overview of large-scale fabric defect detection studies in Section 2.2. Finally, we summarize the publicly available fabric defect datasets in Section 2.3.

### 2.1. Post Hoc Adaptation Techniques

It has been shown that it is possible to train supervised [15,17,19,27,28,29] as well as semi-supervised [11] fabric defect detection methods on multi-fabric datasets. However, it has also been shown that the proposed algorithms generalize poorly to fabrics unseen during training [20,21]. We note that a hybrid, two-step defect detection approach consisting of float-point detection followed by rule-based analysis was shown to work even on woven fabrics unseen during training [30], thus giving a contradiction to the above statement. However, said approach requires high-resolution images for float-point detection, increasing its computational complexity. It has furthermore been validated on a small, simple in-house dataset only, and a rule-based analysis may no longer be feasible for complex fabrics.

As a remedy for the poor performance of supervised models on unseen fabrics, generative adversarial networks (GANs) are commonly employed to synthesize defects/anomalies in the unseen fabrics. The synthetic anomalies are subsequently used in conjunction with readily available, defect-free images to either fine-tune supervised methods [20] or train them from scratch [18]. While defect/anomaly synthesis by means of GANs is also used to improve performance at general surface inspection tasks [31,32], GANs are known to be notoriously difficult to train [33], diminishing the applicability of the developed solutions.

Alternatively, it has been proposed to estimate the probability density function (PDF) of unseen fabrics in the latent features of converged fabric AD models by means of Gaussian mixture models (GMMs) [21]. Here, any additional fine-tuning of the underlying model is omitted, and the negative log-likelihood (NLL) of an image under the estimated PDF is subsequently used as the anomaly score. While omitting model fine-tuning vastly reduces change-over costs/times, potential further performance improvements yielded by adapting the model to the unseen fabric at hand are also discarded.

When considering prior work, it becomes apparent that a large focus is put on adapting converged models to new fabrics. We argue that one should instead focus on learning more universally applicable models that generalize better to unseen fabrics in the first place. Developing such models for the binary fabric AD problem is the goal of this work.

### 2.2. Large-Scale Fabric Defect Detection Studies

To the best of our knowledge, only one large-scale fabric defect detection study exists so far [19], which was conducted concurrent to our work. Here, it was shown that (I) ImageNet pre-training improves defect detection performance and that (II) training on multi-fabric datasets reduces defect detection performance for the individual fabrics. We note, however, that all experiments performed in [19] deal with binary anomaly segmentation (AS) instead of the binary AD task assessed here. Moreover, all evaluations were conducted only within the large-scale dataset that was used for training, i.e., potential effects on the generalization to fabrics unseen during training were not assessed at all in [19].

### 2.3. Public Fabric Defect Datasets

A suitable dataset is needed as the basis for our work. When investigating publicly available datasets in Table 1, it becomes apparent that most of them do not suit the needs of our work: They contain either too few fabrics for a meaningful analysis of input distribution shifts (TILDA [34], AFID [35] and HKU-Fabrics [36]) or do not exhibit the label imbalance inherent to the supervised fabric AD setting. GD-stage 2 [37] specifically contains much more anomalies than normal data, even though the inverse would be the case in the typical supervised fabric AD setting. Thereby, not enough data are available to sample the normal, i.e., defect-free, distribution properly. While both the ZJU-Leaper [19] and the LFLP [17] datasets could be used in theory, they are the result of concurrent work that was not yet publicly available at the time the research presented here was conducted. In addition, only a small validation subset of the LFLP dataset without the bounding boxes is available to the public currently. Moreover, ZJU-Leaper spans only 19 fabrics (as opposed to the 38 fabrics sampled by us, refer to Section 3), reducing the general applicability of any findings. Furthermore, ZJU-Leaper, GD-stage 2 and LFLP only offer limited resolution, which renders the detection of small and subtle defects/anomalies difficult [38]. Last, all publicly available datasets exclusively use front-light illumination. This limits the validity of generated insights, as different defects are detected best in different lighting conditions (this includes both illuminant position and its chosen wavelength spectrum) [39,40,41]. We thus collected and annotated our own large-scale dataset, the details of which will be presented in the following section.

## 3. OLP Dataset

**Figure 2:**
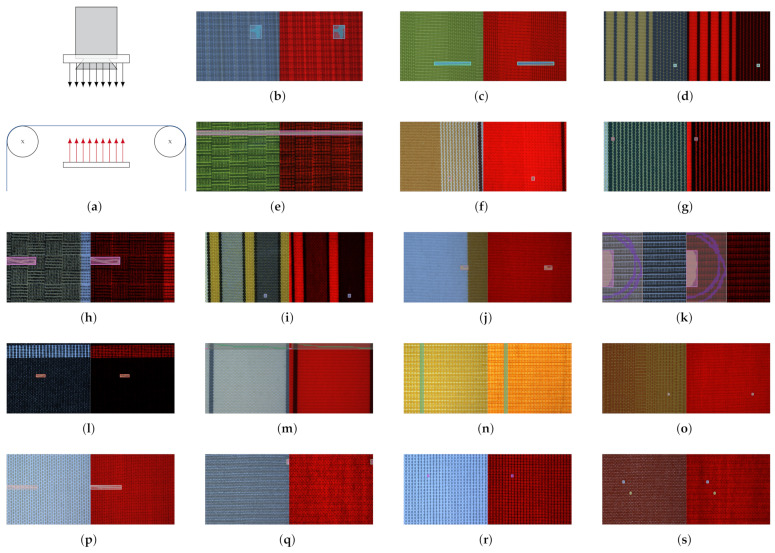
Dataset overview. An outline of the image acquisition setup is shown in (**a**), where blue denotes the inspected fabric specimen, red arrows denote the back-light illumination and black arrows denote the front-light illumination. Representative, anomalous front/back-light image-pairs of fabrics 1–18 with overlaid defect instances (bounding box and mask) are shown in (**b**–**s**).

**Figure 3:**
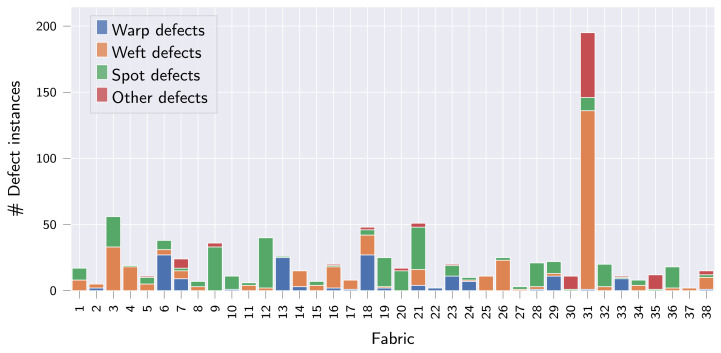
Class distribution of defect instances in the OLP dataset.

While collecting the fabric dataset, focus was put on sampling as many individual fabrics as possible. The final fabric dataset thus comprises a total of 38 woven, patterned fabrics and is named OLP (short for OnLoomPattern, the research grant that funded the dataset acquisition). For each fabric, front-light and back-light RGB image pairs were captured at 2000 dots per inch (DPI) resolution, giving complementary information on light reflectance vs. light transmission of the inspected fabric specimen (refer also to Figure 2a). While a white ring-LED was used for front-light illumination, both red and white LEDs were used for the acquisition of back-light images. In total, the dataset contains 6469 image pairs across all fabrics, of which 627 are labeled as anomalous (see Table 2 and Figure 3 for detailed statistics). For each anomalous image pair, all defect instances were subsequently annotated manually by a single operator, providing both bounding box, segmentation mask and defect type per defect instance. For the classification of defect instances into different defect types, we follow the hierarchical approach from [1], and provide the classification into the following four first-order defect classes: (I) warp defects, (II) weft defects, (III) spot defects and (IV) other defects. A more fine-grained classification of defects as proposed in [42,43] and used in [35,37] is certainly possible but infeasible for the OLP dataset given the limited number of anomalous samples available. Due to the provided defect classification, the dataset is also suited to study the generalization of segmentation and object detection algorithms in the multi-class setting, which the ZJU-Leaper and LFLP datasets cannot be used for. A representative anomalous sample with bounding box and segmentation mask is shown for fabrics 1–18 in Figure 2 to provide a better overview of the dataset.

## 4. Methods

Out of the mechanisms used to explain a model’s failure to generalize, we believe that fabric AD models are affected the most by the “distribution gap” [44], i.e., when training and test data do not originate from the same data distribution. This becomes apparent when considering changes in background fabric appearance as shifts in the underlying data distribution (refer to Figure 1 and Figure 2). Therefore, we argue that factors and techniques which have been shown to improve the robustness of models to data distribution shifts should also increase the generalization of supervised fabric AD methods to unseen fabrics. It should be noted that developing such techniques is in itself an active avenue of research [22]. Moreover, it has been shown recently that the effects of identified factors/techniques are not consistent across different kinds of distribution shifts [22], further increasing the need for our work.

For simplicity, we focus on tried-and-true methods in our evaluations, which we adapt to the supervised fabric AD use-case. Specifically, we (I) make use of VRM, where the vicinity of the training data is sampled, e.g., by means of data augmentation. In addition to VRM, we also assess the following four components that have been reported to influence the resistance of models to distribution shifts [22,23,24,25]: (II) ImageNet pre-training, (III) loss functions, (IV) dataset diversity, as well as (V) model architecture and complexity. We will give the details of each component in the following sections.

### 4.1. Vicinal Risk Minimization

For VRM, we employ rule-based augmentation schemes [13,45,46,47], which require less computation than approaches where the optimal augmentations are learned, e.g., by means of reinforcement learning or adversarial training [22,48,49]. Simultaneously, rule-based augmentation schemes achieve comparable performance if configured properly [22]. Specifically, we adapt AugMix [45], CutOut [47] and MixUp [13] to the supervised fabric AD task and compare their respective influences in Section 5.2.2.

#### 4.1.1. AugMix

**Figure 4:**
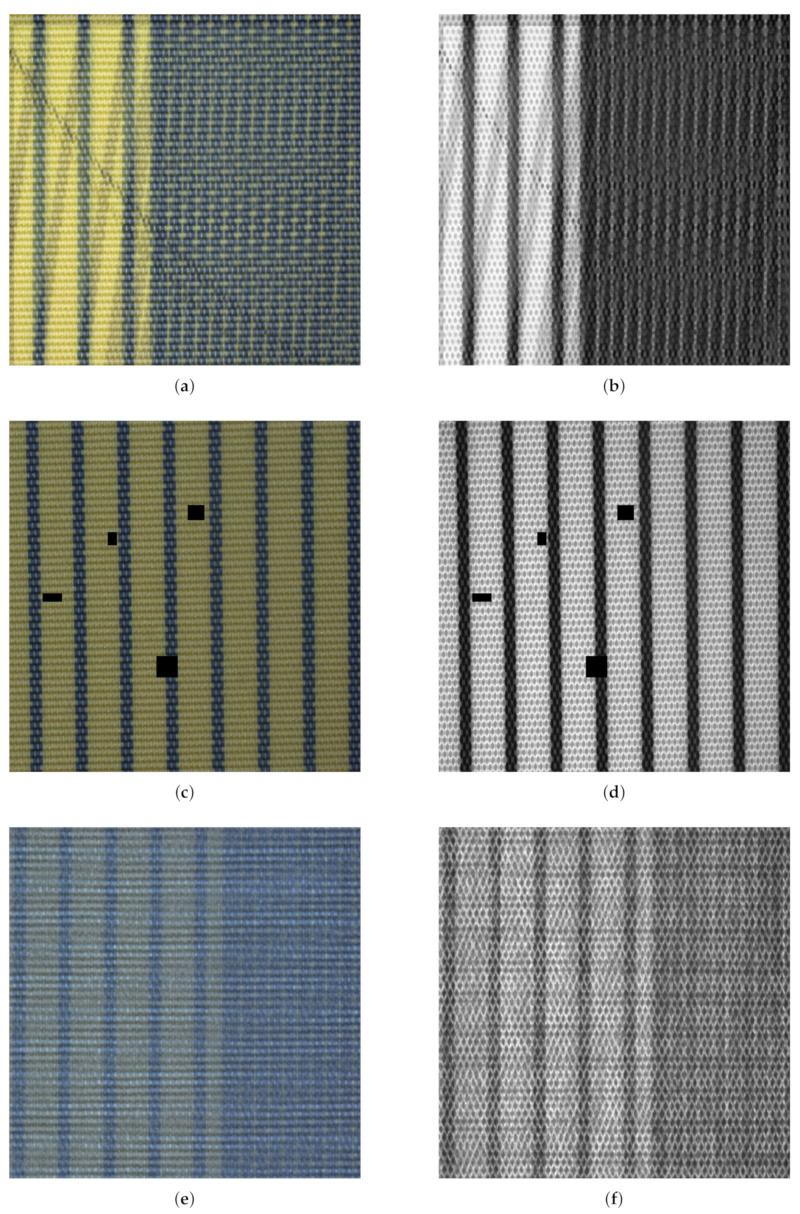
Representative results of applying the three different VRM schemes AugMix (**a**,**b**), CutOut (**c**,**d**) as well as MixUp (**e**,**f**). For the back-light images (**b**,**d**,**f**), the “red” color channel is displayed as a grayscale image, encoding light transmission, whereas light reflectance is shown as RGB images (**a**,**c**,**e**).

In AugMix [45], augmentations are achieved by randomly sampling and compositing augmentations from a predefined set of base augmentations. We adapt AugMix to the fabric AD task by ensuring that every front- and back-light image pair is augmented identically (refer Section 3). Furthermore, we restrict the sampled augmentations and their parametrization to useful values as determined by prior experiments and provide details in Table 3. We leave the parameters α, *width* and *depth* of AugMix at default values, as proposed in [45], and apply AugMix to 50% of the training samples. Last, we omit the Jensen–Shannon divergence term from the augmented images, as it showed no additional benefits during preliminary experiments. A representative sample for the augmentations generated by AugMix is shown in Figure 4a,b.

#### 4.1.2. CutOut

In CutOut [47], random parts from an image are erased in order to enforce that the model bases its decision on multiple features/regions of interest. We adapted CutOut to the fabric AD task by ensuring that the same parts are erased in every front- and back-light image pair. When employed, we apply CutOut to 50% of the training samples and randomly erase between 1 and 4 rectangular patches of width and height ∈[16,64], respectively. These parameters were again determined based on preliminary experiments and remain fixed throughout this work. A representative sample for the augmentations generated by CutOut is shown in Figure 4c,d.

#### 4.1.3. MixUp

In MixUp [13], the vicinity of the training data was sampled by mixing both training samples and their respective class labels. We adapted MixUp to the fabric AD task by ensuring that we only mix within the anomalous and normal samples respectively. The reason for this is that the evaluated hypersphere classifier (HSC) loss (refer Section 4.3.2) requires binary labels, which would no longer be present when mixing between anomalous and normal samples. Apart from this, we left all hyperparameter values of MixUp as proposed in [13] and applied MixUp to all training samples. A representative sample for the augmentations generated by MixUp is shown in Figure 4e,f.

### 4.2. ImageNet Pre-Training

ImageNet pre-training has been shown to improve robustness to data distribution shifts [22,25], and we therefore investigated its potential benefits. Since our input data consisted of effectively 4 color channels (RGB reflectance + transmission luminance, refer Section 5.1), we initialized all but the first convolution layer of the assessed convolutional neural networks (CNNs) with weights generated by training on ImageNet. Thus, the first convolution layer was trained from scratch after being initialized as proposed in [50], whereas the rest of the CNN’s weights were fine-tuned.

The benefits of pre-training have been furthermore reported to diminish with the increasing semantic distance between the target domain and the domain used for pre-training [23,51], which is large in our case. However, these results refer to in-distribution performance only, and it has not yet been investigated whether the same tendency holds also for distribution shifts. Moreover, transfer learning with ImageNet-pre-trained weights was shown to be beneficial for supervised fabric AS based on front-light RGB images recently [19].

### 4.3. Loss Functions

We also evaluated the potential benefits of different loss functions on the generalization of supervised fabric AD methods to unseen fabrics. Specifically, we employed the standard binary cross-entropy (BCE) [52], as well as the HSC [53] and the focal loss (FL) objectives [54]. Both HSC and FL objectives are modifications of the BCE.

#### 4.3.1. BCE

The BCE is defined as:

(1) BCE=−ylog(ϕ(x;W))−(1−y)log(1−ϕ(x;W)),

where ϕ denotes a neural network parametrized by W applied to an image x, and *y* denotes whether an image is considered normal (y=0) or anomalous (y=1).

#### 4.3.2. HSC

Since the BCE does not enforce that normal/defect-free data are concentrated, the HSC objective has been proposed [53], defined as

(2) $$\begin{array}{cc} \mathrm{HSC}= & \left(1-y\right){||\phi (x;W)||}^{2}\\ \; & -y\log\left(1-\exp(-{||\phi (x;W)||}^{2}\right). \end{array}$$

Here, the anomaly score of an image x is given as ${||\phi (x;W)||}^{2}$.

#### 4.3.3. FL

When framing AD to be an imbalanced classification problem, the FL objective can be applied [54]. It modifies the BCE objective to give an increased weight to samples that are currently uncertain/misclassified under the learned decision boundary. Specifically, it is defined as:

(3) $$\begin{array}{cc} \mathrm{FL}= & -y{\left(1-\phi \left(x;W\right)\right)}^{\gamma }\log\left(\phi \left(x;W\right)\right)\\ \; & -\left(1-y\right)\phi {\left(x;W\right)}^{\gamma }\log\left(1-\phi \left(x;W\right)\right), \end{array}$$

with γ being the focusing parameter that can be used to put increasing focus on misclassified samples. During our experiments, we left γ=2 as proposed in [54].

### 4.4. Dataset Diversity

The appearance of fabrics is mainly influenced by the imaging setup and by the fabric properties, which are themselves determined by the material composition (e.g., color) and fabric production parameters (e.g., the weave-repeat for woven fabrics). We argue that our imaging setup is optimal for visual inspection, since it covers both light reflectance and light transmission (refer Figure 2a), the two most important optical properties for fabric defect detection [40]; therefore, we left it fixed throughout our studies. Still, we varied the fabric appearance by acquiring fabrics composed of different materials as well as production properties (refer Figure 2). However, it should be noted that we did not capture images of highly complex fabrics, e.g., fabrics woven via jacquard, and mainly limited ourselves to fabrics composed of synthetic materials. The reason for this is that the majority of technical fabrics, which are subject to stricter quality control, are made from synthetic fibers. To test the influence of dataset diversity on model generalization, we evaluated both a small subset comprised of the 21 first fabrics (referred to as dataset A) and the complete dataset (referred to as dataset B).

### 4.5. Model Architecture and Complexity

We also assessed the influence of model architecture and complexity on the generalization to unseen fabrics. To this end, we trained different variants of the ResNet [55], as well as the EfficientNet [56] architecture. We chose the ResNet since it is a commonly used model architecture in computer vision research and the EfficientNet for its superior performance on ImageNet. This is important, as architectures with better ImageNet performance are more suited for transfer learning [23]. Specifically, we trained ResNet-18, ResNet-34 and ResNet-50, as well as EfficientNet-B0, EfficientNet-B2 and EfficientNet-B4 variants, since initial experiments showed that lower model complexities already achieve competitive/sufficient fabric AD performance. An overview of the models with respect to number of trainable parameters, number of floating-point operations (FLOPs) as well as frames per second (FPS) achieved on an Nvidia RTX 3090 is given in Table 4. We note that the lower FPS for EfficientNet compared to ResNet can be attributed to the unoptimized implementation of depth-wise convolutions in PyTorch [57], the acceleration framework used in our experiments.

### 4.6. Post Hoc Adaptation Methods

We also investigated whether the proposed post hoc adaptation methods benefit from better generalizing models. Specifically, we made use of the procedure proposed in [21] and fit GMMs to estimate the PDF of the unseen fabrics in the latent representations at layer *l* of a converged model $\phi_{l}$. The GMM is defined as:

(4) $$p\left(\phi_{l}\left(x;W\right)\right)=\sum_{i=1}^{K}\psi_{i}N\left(\phi_{l}\left(x;W\right)|\mu_{i},\Sigma_{i}\right),$$

with $\sum_{i=1}^{K}\psi =1$, *K* being the number of Gaussian mixture components and $\mu_{i}$ and $\Sigma_{i}$ denoting the mean vector and covariance matrix of mixture component *i*. We approximate the parameters of the GMM by the expectation maximization (EM) algorithm, as is common practice [58]. We further estimate the number of Gaussian mixture components *K* by using the Bayesian information criterion (BIC), choosing it for its strong regularization characteristics [59]. Moreover, we set *l* equal to the layer used for the HSC objective (i.e., the last feature layer).

Following [21], we used the NLL of the unseen fabric under the estimated PDF,

(5) $$NLL=-log\left(p\left(\phi_{l}\left(x;W\right)\right)\right),$$

as the anomaly score.

While we also tried to evaluate potential benefits yielded by fine-tuning the converged models using normal data of the unseen fabrics, initial experiments showed drastic drops in performance due to the onset of catastrophic forgetting. Furthermore, similar observations have been made for AD in natural images recently [60]. Therefore, we instead evaluated models that have been re-trained from scratch, incorporating normal data of the unseen fabrics here, but note that this is infeasible for an eventual industrial application.

We furthermore failed to assess the benefits of better generalizing models for post hoc adaptation methods that synthesize defects by means of GANs [18,20]. While we did implement them, the GAN-based methods failed to converge for our dataset, due to their complex and inherently unstable training process [33].

## 5. Experiments and Results

We provide a detailed description of general training and evaluation details in Section 5.1. Afterwards, we perform two experiments: First, we analyze how the factors and methods presented above influence the generalization of supervised fabric AD models to both seen and unseen fabrics in Section 5.2. Second, we perform an experiment to test whether models that generalize better are synergetic to post hoc adaptation schemes in Section 5.3.

### 5.1. Evaluation and Implementation Details

Lacking universally applicable measures of model generalization [61,62], we pursue empirical evaluations instead and argue that AD performance should correlate with a model’s ability to generalize. To measure AD performance, we report the area under the receiver operating characteristic (ROC) curve (AUROC) as well as the area under the precision-recall (PR) curve (AUPR). Note that the AUPR is better suited for imbalanced datasets such as ours [63]. In general, we make use of the same training and evaluation strategy as proposed in [21]. Specifically, we employ a leave-one-out (LOO) manner, where all fabrics (except the one that is being evaluated) are used for training large-scale models. The held-out fabric is subsequently used for testing, and the achieved AUPR/AUROC values serve as indicators of a model’s ability to generalize to unseen fabrics. To further increase the statistical robustness, we perform a five-fold evaluation over the fabrics used for large-scale training per held-out fabric. Additionally, a five-fold evaluation is performed on the held-out fabric in a semi-supervised manner, where only the normal images are iterated over. This gives a total of 25 values per held out fabric, and the overall performance is subsequently given by aggregating them, where we report the median *M*, μ as well as σ. In addition to the LOO performance, we also evaluate the performance at the large-scale dataset used for training, extracting a 20% test set from each large-scale dataset, reporting the same aggregated metrics as above. Note that we perform the above evaluations only for fabrics with ≥5 anomalous images, and therefore exclude fabrics 22, 27 and 37 from our evaluations (These fabrics are still included in the large-scale dataset used for model training).

The Adam [64] optimizer is employed for all experiments in combination with the OneCycleLR learning rate policy and a maximum learning rate of 0.001 [65]. All models are trained for 17,500 iterations in total, and the best-performing model is selected based on the AUPR achieved on a 20% validation set extracted from every large-scale dataset. Images are resized to a size of 896 × 896 px, and patch-wise training is performed, where patches of sizes 380 × 380, 260 × 260 and 224 × 224 px are extracted for EfficientNet-B4, EfficientNet-B2 and all other CNN architectures, respectively. Patch sizes correspond to the image sizes used for pre-training the respective models on ImageNet. Conversely to the patch size, EfficientNet-B4 is trained with a batch size of 12, EfficientNet-B2 with a batch size of 14 and all other models with a batch size of 16, which was necessary to facilitate the training of models on hardware with 11 GB VRAM. If anomalies are present in the samples, patches are cropped around them, and randomly otherwise. Furthermore, random oversampling ensures that 25% of training samples are anomalous. Inference is then performed on the whole image, and patch-wise predictions are averaged spatially to yield image-level predictions.

### 5.2. Improving the Performance of Supervised Fabric AD Methods on Unseen Fabrics

We note that we do not evaluate every single possible permutation of the hyperparameters but instead limit ourselves to useful combinations in each of the following sections. This was necessary to reduce the required computation for the experiments to a feasible amount: we trained 6720 models as opposed to the >40,000 permutations possible, and training a single model took between 2 and 4 h on a modern GPU.

#### 5.2.1. Effects of Pre-Training, Loss Function and AugMix

We begin by jointly assessing effects of pre-training, loss function as well as AugMix on the performance on previously unseen fabrics, fixing the dataset composition to dataset B and the model to an EfficientNet-B0.

**Figure 5:**
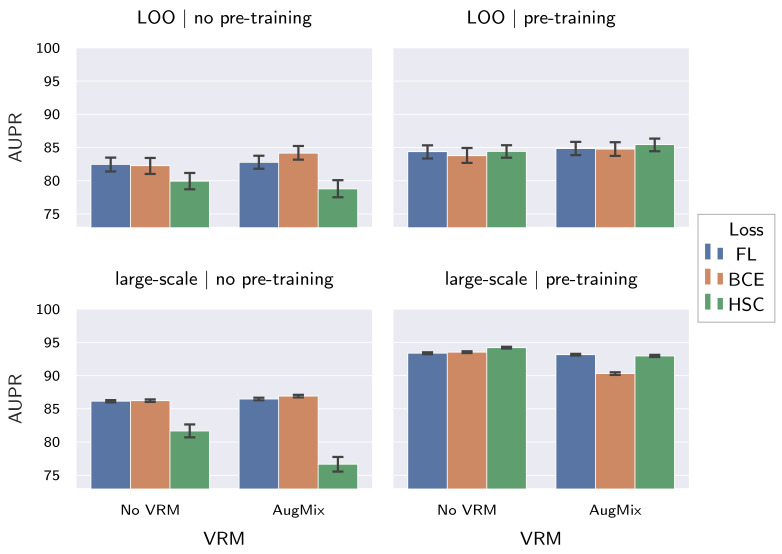
Influence of ImageNet pre-training, loss function and AugMix on the resistance of supervised fabric AD methods to distribution shifts. We show both mean and 95% confidence interval (CI) for the AUPR achieved on the held-out fabrics, as well as on the large-scale dataset.

Regarding the three tested components, the results in Table 5 and Figure 5 show the following: (I) Pre-training on ImageNet improves AD performance on both the large-scale dataset and on the unseen LOO fabrics, as denoted by increased AUPR and AUROC values. Moreover, effects are larger for AUPR compared to AUROC. Combined with the fact that AUPR is the better evaluation measure for imbalanced datasets such as ours, this indicates a significant performance increase for the large-scale dataset. (II) Loss functions on their own perform comparable to each other. It should be noted, however, that training with the HSC loss benefits the most from pre-training, as well as from AugMix, and thus performs the best overall. Moreover, the large values observed for σ when training from scratch with the HSC loss on the large-scale dataset indicate instable convergence, which was confirmed manually. (III) VRM, by means of AugMix, generally improves AD performance in unseen fabrics, while simultaneously reducing the performance in the large-scale dataset used for training. This is especially true when pre-training on ImageNet is applied. Therefore, large-scale dataset performance is not necessarily indicative of LOO performance, further demonstrating the need for our research.

##### 5.2.1.1. Variance Decomposition

**Figure 6:**
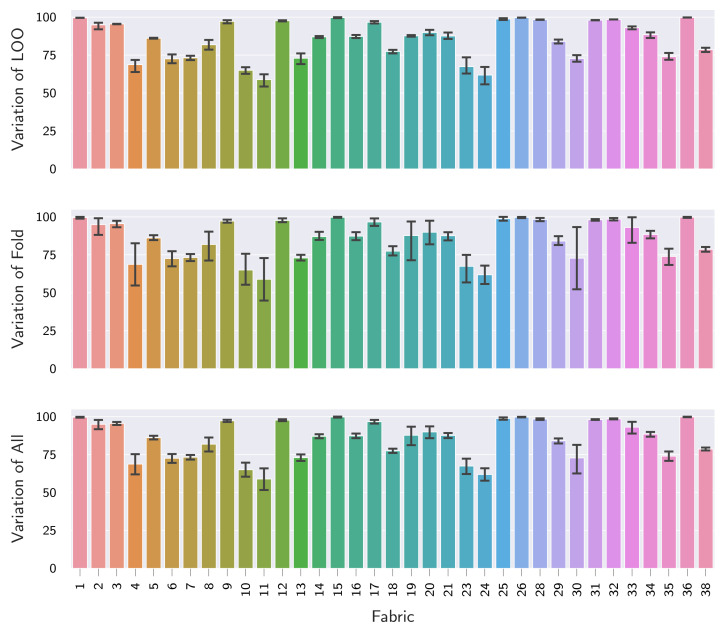
Variance decomposition of the LOO performance for an ImageNet pre-trained EfficientNet-B0 when combined with HSC loss and the application of AugMix. We show both mean and 95% CI for the AUPR on the unseen fabrics for all data points (Variation of All), as well as when first aggregating over the five LOO folds (Variation of Fold) and the five large-scale dataset folds (Variation of LOO), respectively.

Moreover, it can be seen that LOO performance varies strongly across all assessed configurations, as given by the large values for σ in Table 5. We therefore perform a variance decomposition next and show bar plots for the LOO performance of the currently best performing configuration, an EfficientNet-B0 pre-trained on ImageNet combined with HSC loss and the application of AugMix, in Figure 6. Here, we plot μ and its 95% CI estimated from the 25 values of each respective fabric. In addition to the overall variation, we also show μ and 95% CIs when first aggregating over the five LOO folds (Variation of Fold) and the five large-scale dataset folds (Variation of LOO), respectively.

Assessing the results in Figure 6, it can be seen that the LOO fabric has the biggest influence, as indicated by the difference in μ AUPR across fabrics. Furthermore, it can also be seen that additional variance is introduced by the large-scale folds and model training (Variation of Fold). It should be noted that the severity of the incurred variance here again strongly depends on the LOO fabric, as denoted by inconsistent CI sizes across fabrics. Last, it can be seen that the semi-supervised splits over the LOO fabrics themselves have the least influence on LOO performance. This indicates that even a small sample of defect-free images is already sufficient to characterize an unseen fabric well.

##### 5.2.1.2. Analysis of Latent Embeddings

Next, we investigate the topological structure of the learned feature representations to see how they differ between fabrics with high and low LOO performance. We apply the UMAP algorithm [66] to generate 2D embeddings of the EfficientNet-B0’s feature representations for both fabric 12 (a fabric with high LOO performance) and fabric 23 (a fabric with low LOO performance). We chose UMAP over other, competing embedding methods, such as t-distributed stochastic neighbor embedding (t-SNE), as it was shown to preserve the global structure of the original feature representation more accurately [66]. Similar to our evaluation scheme, we apply the UMAP algorithm in the LOO fashion, i.e., we fit it using the test set of the large-scale dataset and project both the test set of the large-scale dataset and the LOO fabrics into the learned embedding.

**Figure 7:**
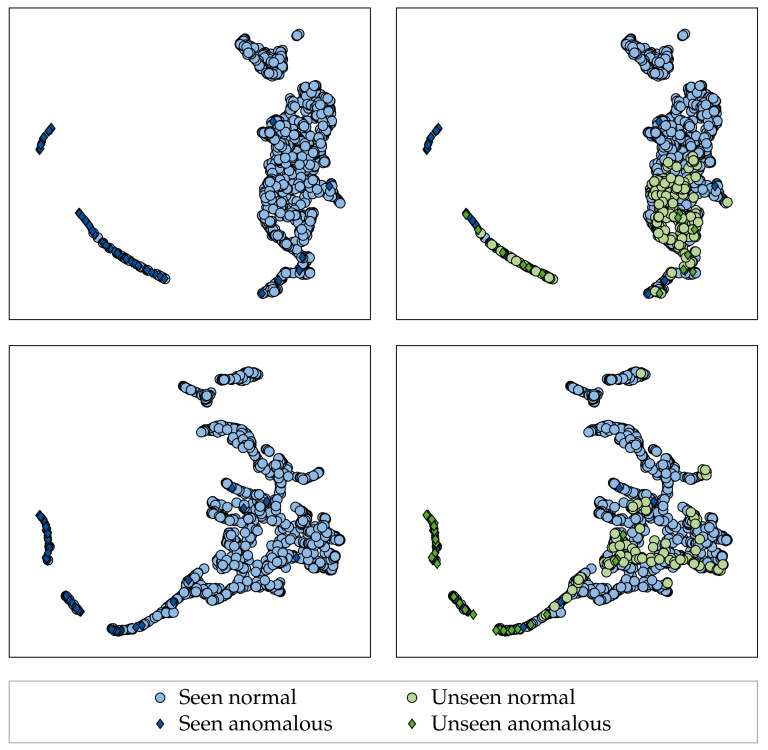
Two-dimensional UMAP embeddings of a pre-trained EfficientNet-B0 fine-tuned with the HSC objective under the application of AugMix in a LOO manner on Dataset B for fabric 23 (top) and fabric 12 (bottom). Left shows the embedding for the test set of the large-scale dataset used for fine-tuning, whereas right additionally shows the held out fabric projected into the same embedding.

Figure 7 shows that anomalies in the large-scale dataset form a distinct cluster away from the normal/defect-free data distribution for both the fabric with high LOO performance and the fabric with low LOO performance. However, it can be seen that the anomalies of the fabric with high LOO performance lie closer to the anomalies of the large-scale dataset compared to the anomalies of the fabric with low LOO performance. Furthermore, defect-free data are more often mapped to the anomaly-cluster for the fabric with low LOO performance than for the fabric with high LOO performance. This indicates that the AD performance on previously unseen fabrics is affected by shifts in both the normal and the anomaly distribution.

#### 5.2.2. Effects of Dataset Composition and VRM Type

We continue by investigating the effects of dataset composition and choice of VRM type on the generalization to previously unseen fabrics. We fix the model to an EfficientNet-B0 pre-trained on ImageNet and the loss function to HSC, due to their superior performances in the prior experiment.

**Figure 8:**
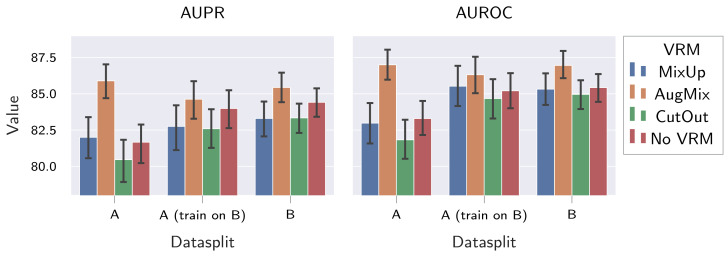
Influence of dataset composition and VRM type on the performance of supervised fabric AD methods on unseen fabrics. We show both mean and 95% CI for the AUPR and the AUROC achieved on the unseen fabrics by a pre-trained EfficientNet-B0 fine-tuned with the HSC loss.

Results in Table 6 and Figure 8 show the following: (I) More diverse datasets benefit the performance on previously unseen fabrics, especially when VRM is omitted, as indicated by an increase in AUPR as well as AUROC. Furthermore, more diverse datasets also slightly improve consistency across fabrics, as denoted by a lower σ for AUPR on dataset A when large-scale training is performed on dataset B compared to training and evaluating on dataset A. (II) Not all VRM schemes increase LOO performance. In fact, CutOut even decreases LOO performance compared to the baseline (omitting VRM), as indicated by lower AUPR and AUROC values. On the other hand, inconsistent effects are observed for MixUp, where *M* AUPR and AUROC scores are improved, but simultaneously, σ vastly increased and μ is slightly reduced. Out of the investigated VRM schemes, only AugMix consistently improves LOO performance. However, it should be noted that its benefits are larger for dataset A than for dataset B. Moreover, AugMix combined with training on dataset B perform worse on fabrics of dataset A compared to training on dataset A exclusively under the application of AugMix. Still, AugMix improves LOO performance, even in this setting.

Based on the above findings, we restrict all further experiments to the larger dataset B and only contrast AugMix with the omission of VRM.

#### 5.2.3. Effects of Model Architecture and Complexity

Next, we investigate the effects of model architecture and complexity on the generalization of supervised fabric AD methods. We fix the loss to HSC, train with or without AugMix, and only use models pre-trained on ImageNet.

**Figure 9:**
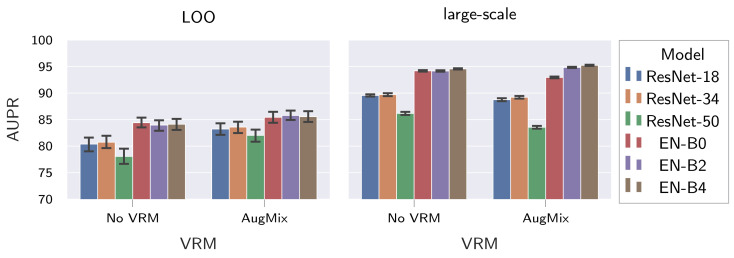
Influence of model architecture and complexity as well as AugMix on the generalization of supervised fabric AD methods. We show both the mean and 95% CI for the AUPR achieved on the held-out fabrics, as well as on the large-scale dataset by different ImageNet-pre-trained models that are fine-tuned with the HSC loss. Abbreviations: EN = EfficientNet.

The results in Table 7 and Figure 9 show the following: (I) Model architectures with better ImageNet performance also achieve better supervised fabric AD performance, both on the test set of the large-scale dataset and on unseen, held-out fabrics. This can be inferred from the fact that EfficientNets outperform ResNets on both tasks in question. (II) No clear tendencies can be observed for the influence of model complexity. While the large-scale dataset performance tends to increase for more complex EfficientNet variants, and the best performance is achieved by the EfficientNet-B4, the best LOO performance is achieved by the EfficientNet-B2. For the ResNet architecture, on the other hand, the best large-scale dataset performance as well as LOO performance is achieved by the ResNet-34. Thus, more complex models do not necessarily have an improved resistance to input distribution shifts for supervised fabric AD. (III) VRM by means of AugMix is beneficial for the LOO performance of all investigated models. It should be noted, however, that no clear influence of AugMix on large-scale dataset performance can be observed any longer (compare Table 7 with Table 5).

Based on the above findings, we limit further evaluations to models of the EfficientNet architecture, as they showed better performance both on the large-scale dataset and on the unseen fabrics.

### 5.3. Do Post Hoc Adaptation Methods Also Benefit from Models That Generalize Better?

In this section, we test whether models that generalize better are synergetic to post hoc adaptation methods. Since post hoc adaptation by means of fine-tuning and GAN-based defect synthesis failed, we re-trained models under the addition of defect-free data from the held-out fabrics for comparison instead.

The results in Table 8 show the following: (I) Post-hoc adaptation by means of PDF-estimation as proposed in [21] improves LOO performance in all assessed configurations. It is therefore synergetic to models that generalize better. (II) Adding the normal data of the held-out fabrics to the large-scale dataset for model re-training surprisingly decreases large-scale performance in all assessed configurations. It is therefore apparently detrimental to the generalization within the dataset itself. However, it improves the performance on the held-out fabrics for all models but EfficientNet-B2 when combined with PDF-estimation by means of GMM and AugMix. (III) Last, a re-training with added defect-free data performs best amongst all evaluated approaches on the unseen fabrics (compare Table 8 with Table 5, Table 6 and Table 7), achieving an *M* AUPR of 91.8, a μ of 87.4, and a σ of 13.6. This demonstrates that there is further room for improvement in increasing the generalization of models to unseen fabrics.

## 6. Discussion

We have investigated the influence of various components on the generalization of supervised fabric AD methods and give a high-level summary of identified trends in Table 9.

Considering the large differences between large-scale dataset performance and performance on the held-out fabrics, e.g., μ±σ AUPR of 95.2±1.7 vs. 85.6±14.7 for an EfficientNet-B4 pre-trained on ImageNet and fine-tuned with HSC loss under application of AugMix on dataset B (refer Table 8), it can be concluded that cross-fabric generalization of supervised fabric AD models is indeed largely affected by the “distribution gap”. This is further supported by the fact that the LOO fabric had the biggest influence on the conducted variance decomposition (refer Section 5.2.1.1). Moreover, when investigating the unseen fabrics in the latent embeddings of the models, it could be seen that a shift occurs in both the normal and the anomaly distribution for fabrics with low LOO performance compared to those with high LOO performance (Section 5.2.1.2). Combined with other recent works [53,67], this indicates that the anomaly distribution does not follow a uniform distribution over the latent space [68,69] but rather follows a mixture distribution. We therefore argue that future work on supervised fabric AD/defect detection should always perform hold-out experiments similar to ours in order to assess the resistance of proposed methods to distribution shifts (as denoted by LOO performance) in addition to the generalization within the distributions used for training (large-scale dataset performance).

With respect to the influence of the individual components assessed in this work, several conclusions can be drawn: First, ImageNet pre-training increases both generalization within the dataset used for training and the resistance to input distribution shifts and should therefore be used whenever possible (Section 5.2.1). We thus observe trends similar to [22,25], even for datasets that differ greatly from natural images in their appearance [23,51], and note that the same observation was made in the medical domain recently [70]. Moreover, ImageNet-weights were only partially transferred in our work, as the first convolution layer was trained from scratch due to the multichannel nature of the employed image-acquisition setup (refer Figure 2a). Here, useful next steps would be to contrast pre-training on different datasets, as performed in [51]. Notably, this comparison should also include defect detection datasets, as a high similarity between source and target domain as well as source and target tasks have been shown to improve transfer learning performance [51].

Next, loss functions perform comparable to each other when employed without pre-training or VRM (Section 5.2.1). However, when combined with AugMix and ImageNet pre-training, HSC loss was shown to slightly outperform the other losses, with respect to both generalization within the large-scale dataset and resistance to input distribution shifts. This can be attributed to the fact that HSC is the only loss out of those evaluated that enforces a clustering of the normal data distribution (refer Section 4.3.2). As this poses a stronger constraint, it therefore seems likely that shifts in the normal data distribution affect models trained with this loss only to a lesser extent.

When investigating the influence of dataset composition and VRM-type (Secton Section 5.2.2), it was shown that more diverse datasets increase the resistance to input distribution shifts. Moreover, good large-scale dataset performance was observed also for the large dataset B. This is in contradiction to [19], where worse large-scale dataset performance was observed for more diverse fabric datasets compared to less-diverse datasets. However, it should be noted that the research focused on AS rather than AD in [19]. We will therefore revalidate our findings on the ZJULeaper dataset [19] in future work. Regarding the different VRM types, it was found that only AugMix consistently improves resistance to distribution shifts. However, AugMix simultaneously reduced the generalization within the large-scale dataset in four of the six evaluated model configurations (Table 7). This indicates that VRM methods proposed on natural images might not be easily transferred to the supervised fabric AD task, and developing schemes that consistently improve both the resistance to distribution shifts and generalization within the large-scale dataset is thus left for future work. Here, we propose to adapt augmentation schemes where the best possible augmentations are learned [22,48,49] to the fabric inspection task or to employ adversarial training with on-manifold adversarial examples [71].

Regarding model architecture and model complexity (Section 5.2.3), it was found that architectures with improved ImageNet performance had both better generalization within the large-scale dataset and stronger resistance to distribution shifts compared to architectures with lower ImageNet performance. Regarding model complexity, no conclusive statement can be made, as best generalization within the large-scale dataset was achieved by EfficientNet-B4, yet highest resistance to input distribution shifts was observed for EfficientNet-B2. This finding is in direct contradiction to the statement that increasing model complexity improves resistance to distribution shifts of input data [72,73]. However, exhaustive evaluations of said hypothesis were inconclusive [22] and, therefore, in agreement with the findings presented here. Thus, the influence of model complexity on the resistance to input distribution shifts most likely depends on the performed task and the used data, and can be either beneficial or detrimental.

We furthermore investigated whether the observed tendencies are synergetic to post hoc adaptation techniques (Section 5.3) and found this to be the case. Since fine-tuning the models and their learned representations in a post hoc manner failed, we chose to re-train all supervised fabric AD methods under the addition of normal data from the held-out fabrics instead. Interestingly, this approach decreased both the generalization within the large-scale dataset and the resistance to input distribution shifts on its own. When coupled with post hoc adaptation by means of PDF-estimation, however, resistance to input distribution shifts was improved. From this, it can be inferred that defect-free samples of a fabric can be used to generate more discriminative feature representations, but that both defect and defect-free samples of a fabric are required in order to learn a discriminative decision boundary in said feature representations. Due to the limited overall performance gains and the large computational cost incurred from re-training the model per fabric, however, this method cannot be recommended for further use. Moreover, the combination of its low performance gains with the observed shifts in the anomaly distribution (Section 5.2.1.2) indicates that successfully developing additional post hoc adaptation methods based on defect-free data only might be difficult.

### Limitations

While we created a large-scale fabric dataset, we limited ourselves to fabrics composed of synthetic materials and medium complexity of appearance. We will therefore focus on further increasing the diversity of our fabric dataset by sampling a wider variety of materials (e.g., cotton) and fabric types (e.g., jacquard fabrics) in future work. Moreover, we focused our evaluations on the OLP fabric dataset created in this work. We will therefore revalidate our findings on the ZJULeaper dataset [19] in future work. Furthermore, we still require labeled anomalies to assess fabric AD performance on unseen fabrics. Instead, it would be preferable to estimate a model’s applicability on previously unseen fabrics using normal data only, which are more readily available. To this end, we will develop measures that use normal data only in future work, basing them on generalization measures [61,62]. In this context, we will also try to quantify the severity of the shifts occurring for both the anomaly and the normal data distribution. Last, we limited our analysis to supervised fabric AD. We will therefore extend our analysis to supervised AS [15,19,74,75] as well as object/defect detection methods [16,28,76] in future work. We note that it would be interesting to assess the resistance of reference-based approaches [76] to input distribution shifts here, given that reference images should provide ample information about the shift in the normal/defect-free data distribution.

## 7. Conclusions

In our work, we hypothesized that the generalization of supervised fabric AD methods to fabrics unseen during training is mostly affected by the “distribution gap”, and confirmed this hypothesis experimentally. Here, results showed that shifts which reduce the performance on unseen fabrics occur both in the anomaly and in the normal data distribution. Investigations into the resistance to these shifts revealed (I) that pre-training on ImageNet is beneficial; (II) that HSC loss outperforms the other losses when combined with ImageNet pre-training and (III) AugMix, which is the only VRM technique that increases the resistance to input distribution shifts consistently; (IV) increasing the dataset diversity is also beneficial on its own; and (V) model architectures with better ImageNet performance also have better resistance to distribution shifts. Moreover, as opposed to the literature, increasing the model complexity was neither beneficial nor detrimental. We expect our work to facilitate the industrial realization of supervised fabric AD methods and will continue to improve both the generalization of supervised fabric AD methods within the large-scale dataset and their resistance to distribution shifts in future work.

## Figures and Tables

**Table 1:** Comparison of publicly available fabric defect datasets and the dataset OLP generated in this study. We use – to denote when the information about a characteristic is not available for a given dataset. Note that ZJU-Leaper [19] and LFLP [17] are concurrent works that were not yet publicly available at the time the research presented in this paper was conducted. Furthermore, ✓ denotes the presence of an attribute, and ✗ denotes its absence. Abbreviations: FL = Front-light; BL = Back-light; M.c.s.l. = Multi-class single-label; # F = Number of fabrics; BB = Bounding box; Anom = Anomalous.

Dataset	Images									Annotations		
	# F	Anom.	Normal	Total	Color	FL	BL	Image Size	dpi	Label	BB	Mask
TILDA [34]	8	2798	400	3198	Gray	✓	✗	768 × 512 px	–	M.c.s.l.	✗	✗
AFID [35]	7	105	140	245	Gray	✓	✗	4096 × 256 px	100	M.c.s.l.	✗	✓
HKU-Fabric [36]	3	81	85	166	Gray	✓	✗	256 × 256 px	200	M.c.s.l.	✗	✓
GD-stage 2 [37]	50	4354	910	5264	RGB	✓	✗	4096 × 1800 px	–	M.c.s.l.	✓	✗
LFLP [17] ^a^	50	25,456	41,124	66,580	RGB	✓	✗	512 × 512 px	–	Binary	✓	✗
ZJU-Leaper [19]	19	27,650	71,127	98,777	RGB	✓	✗	512 × 512 px	–	Binary	✓	✓
OLP (ours)	38	627	5842	6469	RGB	✓	✓	2448 × 2050 px	2000	M.c.s.l.	✓	✓

^a^ Only a limited subset of the dataset without the bounding boxes is publicly available at the time of this work.

**Table 2:** Detailed characteristics of the OLP dataset. Abbreviations: # = Number of.

Fabric	# Images		# Defect Instances			
	Normal	Anomalous	Warp	Weft	Spot	Other
1	470	14	0	8	9	0
2	242	5	2	3	0	0
3	180	44	0	33	23	0
4	229	19	0	18	1	0
5	227	9	0	5	5	1
6	583	23	27	4	7	0
7	513	24	9	6	2	7
8	159	6	0	3	4	0
9	118	26	0	0	33	3
10	78	6	1	0	10	0
11	35	5	0	4	2	0
12	112	35	0	2	38	0
13	95	25	25	0	1	0
14	201	13	3	12	0	0
15	64	7	0	4	3	0
16	305	20	2	16	1	1
17	45	7	1	7	0	0
18	389	45	27	15	4	2
19	55	16	2	1	22	0
20	42	17	0	0	15	2
21	213	39	4	12	32	3
22	58	2	2	0	0	0
23	216	14	11	0	8	1
24	171	9	7	1	2	0
25	52	8	0	11	0	0
26	39	21	0	23	2	0
27	42	3	0	1	2	0
28	99	21	1	2	18	0
29	104	16	11	2	9	0
30	49	11	0	0	1	10
31	146	34	1	135	10	49
32	40	20	0	3	17	0
33	49	11	9	0	1	1
34	52	8	0	4	4	0
35	50	10	0	0	1	11
36	36	18	0	2	16	0
37	58	2	0	2	0	0
38	226	14	1	9	2	3
Total	5842	627	146	348	305	94

**Table 3:** Base augmentations and their parametrization range employed for AugMix.

Augmentation	Hyperparameter Values
Autocontrast	-
Equalize	-
Posterize	bits∈{3,4}
Solarize	threshold=77
Rotation	$\mathrm{angle}\in [-5^{\circ },5^{\circ }]$
Translation	$\Delta_{x},\Delta_{y}\in \left[-0.0625,0.0625\right]$
Shear x	angle∈[−0.09,0.09]
Shear y	angle∈[−0.09,0.09]

**Table 4:** Architecture specifications for ResNet and EfficientNet.

Model	Parameters	FLOPs	FPS
ResNet-18	11.7 M	1.8 G	1926.0
ResNet-34	21.8 M	3.6 G	1392.3
ResNet-50	25.6 M	3.8 G	756.6
EfficientNet-B0	5.3 M	0.39 G	749.8
EfficientNet-B2	9.2 M	1.0 G	543.9
EfficientNet-B4	19 M	4.2 G	193.3

**Table 5:** Influence of ImageNet pre-training, loss function and AugMix on the resistance of supervised fabric AD methods to distribution shifts. Best scores for transfer learning and training from scratch are highlighted in bold.

Pre- Training	Loss	VRM	AUPR						AUROC					
			LOO			Large-Scale			LOO			Large-Scale		
			M	μ	σ	M	μ	σ	M	μ	σ	M	μ	σ
✗	BCE	✗	87.2	82.3	17.6	86.0	86.2	2.9	89.8	85.0	16.4	96.5	**96.5**	**0.9**
		AugMix	**88.8**	**84.2**	**15.1**	**87.0**	**86.9**	3.1	89.6	**86.5**	**13.4**	**96.6**	96.4	1.2
	FL	✗	86.3	82.5	16.5	86.4	86.1	**2.7**	**90.0**	85.5	14.5	96.4	96.3	1.0
		AugMix	87.1	82.8	16.0	86.5	86.5	2.9	88.9	85.5	13.6	96.3	96.3	1.1
	HSC	✗	84.5	79.9	18.3	86.9	81.7	15.8	85.4	81.3	16.9	96.3	94.2	7.1
		AugMix	83.5	78.8	19.4	81.6	76.7	15.8	86.2	80.2	18.8	94.3	92.4	6.7
✓	BCE	✗	86.9	83.8	16.3	93.7	93.5	**2.0**	**91.3**	86.2	15.2	**98.1**	**98.0**	**0.9**
		AugMix	87.5	84.8	14.8	90.6	90.3	3.0	90.4	**87.2**	14.3	96.8	96.7	1.5
	FL	✗	87.9	84.4	15.2	93.6	93.4	2.2	90.0	86.2	15.1	97.9	97.7	1.1
		AugMix	87.3	84.8	**14.5**	93.3	93.1	**2.0**	91.1	87.1	**13.7**	97.9	97.8	**0.9**
	HSC	✗	87.0	84.4	14.7	**94.6**	**94.2**	2.1	88.9	85.4	14.5	**98.1**	97.9	1.2
		AugMix	**89.4**	**85.4**	15.1	93.1	92.9	2.4	90.9	87.0	14.5	97.7	97.5	1.5

**Table 6:** Influence of dataset composition and VRM type on the performance of supervised fabric AD methods on unseen fabrics. Scores on the held-out fabrics are reported for a pre-trained EfficientNet-B0 fine-tuned with the HSC loss. Best scores within each dataset are highlighted in bold.

Dataset	VRM	AUPR			AUROC		
		M	μ	σ	M	μ	σ
A	✗	86.0	81.7	15.8	85.7	83.3	14.1
	AugMix	**89.5**	**85.9**	**13.4**	**90.1**	**87.0**	**12.4**
	CutOut	83.4	80.5	16.7	84.1	81.8	15.7
	MixUp	86.6	82.0	17.2	87.3	83.0	16.6
A (train on B)	✗	86.5	84.0	**14.3**	89.0	85.2	**14.5**
	AugMix	87.4	**84.6**	14.7	90.2	**86.3**	14.9
	CutOut	86.0	82.6	15.4	89.0	84.7	15.3
	MixUp	**88.3**	82.8	18.3	**90.6**	85.5	16.0
B	✗	87.0	84.4	**14.7**	88.9	85.4	**14.5**
	AugMix	**89.4**	**85.4**	15.1	**90.9**	**87.0**	**14.5**
	CutOut	86.4	83.3	15.7	89.1	85.0	15.0
	MixUp	88.7	83.3	18.0	90.0	85.3	15.9

**Table 7:** Influence of model architecture and complexity as well as AugMix on the generalization of supervised fabric AD methods. Scores on the held-out fabrics and on the test set of the large-scale dataset used for model training are reported for pre-trained models fine-tuned with HSC loss and with or without application of AugMix. Dataset B is used for training, and best scores within each model architecture are highlighted in bold.

Architecture	VRM	AUPR						AUROC					
		LOO			Large-Scale			LOO			Large-Scale		
		M	μ	σ	M	μ	σ	M	μ	σ	M	μ	σ
EfficientNet-B0	✗	87.0	84.4	14.7	94.6	94.2	2.1	88.9	85.4	14.5	98.1	97.9	1.2
	AugMix	89.4	85.4	15.1	93.2	93.0	2.4	90.9	**87.0**	14.5	97.8	97.5	1.5
EfficientNet-B2	✗	86.3	84.0	14.7	94.5	94.2	2.2	87.7	84.5	15.2	97.9	97.7	1.2
	AugMix	**90.1**	**85.8**	**13.8**	95.2	94.8	1.8	89.8	**87.0**	**12.3**	98.3	98.1	1.0
EfficientNet-B4	✗	87.9	84.1	15.5	94.6	94.6	**1.7**	89.0	84.5	16.0	98.0	97.9	1.0
	AugMix	90.0	85.6	14.7	**95.4**	**95.2**	**1.7**	**91.5**	86.8	14.3	**98.4**	**98.3**	**0.9**
ResNet-18	✗	85.5	80.4	19.2	89.2	89.6	**3.5**	88.7	84.1	15.5	97.5	97.4	1.0
	AugMix	86.2	83.2	**16.1**	89.0	88.8	4.1	89.8	86.2	**13.3**	97.4	97.3	1.0
ResNet-34	✗	84.0	80.7	17.7	**90.3**	**89.7**	4.3	87.7	84.1	15.1	97.7	97.4	1.1
	AugMix	**88.1**	**83.6**	16.2	89.7	89.2	3.7	**90.9**	**86.6**	14.0	**97.6**	**97.5**	**0.9**
ResNet-50	✗	84.2	78.1	20.9	86.7	86.2	4.6	87.5	82.7	17.5	97.2	97.1	1.1
	AugMix	86.3	82.0	17.4	84.1	83.6	3.9	90.3	84.6	15.6	96.8	96.6	1.1

**Table 8:** Are models that generalize better synergetic to post hoc adaptation methods? Scores on the held-out fabrics and on the test set of the large-scale dataset used for model training are reported for pre-trained models fine-tuned with HSC loss and with or without application of AugMix. Furthermore, models are trained with or without addition of normal data from the held-out fabric to the large-scale dataset used for training (N). In addition to the performance of the learned decision boundary on the held-out fabric (LOO), we also report the performance of the model when subjected to post hoc adaptation as proposed in [21] (PDF). Dataset B is used for training, and best scores within each model are highlighted in bold. Abbreviations: EN = EfficientNet; A = AugMix.

EN	A	N	AUPR									AUROC								
			LOO			PDF			Large-Scale			LOO			PDF			Large-Scale		
			M	μ	σ	M	μ	σ	M	μ	σ	M	μ	σ	M	μ	σ	M	μ	σ
B0	✗	✗	87.0	84.4	**14.7**	89.2	85.5	14.4	**94.6**	**94.2**	**2.1**	88.9	85.4	14.5	93.0	87.6	13.7	**98.1**	**97.9**	**1.2**
		✓	84.7	81.4	17.2	89.9	86.6	14.1	91.2	90.6	3.9	82.9	80.8	17.3	92.3	88.5	13.3	95.9	95.1	3.1
	✓	✗	**89.4**	**85.4**	15.1	88.8	85.8	15.3	93.2	93.0	2.4	**90.9**	**87.0**	**14.5**	93.0	**88.8**	**13.0**	97.8	97.5	1.5
		✓	86.0	82.5	16.7	**90.4**	**87.1**	**13.6**	91.2	90.7	3.7	86.4	81.4	17.9	**93.1**	88.7	13.3	95.9	95.3	3.0
B2	✗	✗	86.3	84.0	14.7	87.5	84.7	15.0	94.5	94.2	2.2	87.7	84.5	15.2	92.1	87.1	14.2	97.9	97.7	1.2
		✓	82.4	81.5	16.3	90.7	86.8	13.8	91.5	90.8	4.0	84.2	80.8	16.4	92.2	88.8	12.4	95.6	95.1	3.1
	✓	✗	**90.1**	**85.8**	**13.8**	**90.9**	**87.4**	**12.8**	**95.2**	**94.8**	**1.8**	**89.8**	**87.0**	**12.3**	**93.4**	**90.1**	**11.1**	**98.3**	**98.1**	**1.0**
		✓	87.8	83.4	16.6	**90.9**	87.1	13.8	92.3	91.4	3.6	87.9	83.5	15.8	93.3	89.1	12.4	96.4	95.6	2.8
B4	✗	✗	87.9	84.1	15.5	88.1	85.0	15.1	94.6	94.6	**1.7**	89.0	84.5	16.0	91.9	87.0	15.0	98.0	97.9	1.0
		✓	86.7	83.1	16.4	89.7	85.8	14.8	92.2	91.4	3.9	87.8	83.2	16.3	92.9	87.0	15.0	96.3	95.7	2.9
	✓	✗	**90.0**	**85.6**	**14.7**	89.9	85.9	15.0	**95.4**	**95.2**	**1.7**	**91.5**	**86.8**	**14.3**	93.2	88.0	14.3	**98.4**	**98.3**	**0.9**
		✓	89.9	84.5	15.5	**91.8**	**87.4**	**13.6**	92.7	91.9	3.5	88.1	84.8	15.0	**94.3**	**89.0**	**13.6**	96.7	95.9	2.7

**Table 9:** High-level summary of trends identified for the generalization of supervised fabric AD methods. We denote positive influence of methods with ↑, and negative as well as inconclusive influence with –.

Method	Generalization to	
	Test Data	Unseen Fabrics
ImageNet pre-training	↑	↑
VRM techniques	–	–
Larger/more complex models	–	–
Better model architectures ^a^	↑	↑
Higher dataset diversity	–	↑
Loss functions	–	–

^a^ As denoted by ImageNet classification performance.

## Data Availability

Publicly available datasets were analyzed in this study. These data can be found here: https://github.com/ORippler/OLP-dataset.
